# False Impressions

**Published:** 1888-12-08

**Authors:** Caroline Fothergill

**Affiliations:** Author of "An Enthusiast," "A Voice in the Wilderness," etc.


### False Impressions.

By Caroline Fothergill, Author of "An Enthusiast," "A Voice in the Wilderness," etc.

*(Continued from p. 142.)*

### Chapter X.—Disappointment.

The conversazione was, if possible, even more enjoyable to Edith than the flower show. The pictures, the brilliantly-lighted rooms, the moving crowds, the beautiful dresses, and the consciousness that she herself was as pretty and well-dressed a girl as there was present, all contributed to raise her spirits, and cause her to see everything in the brightest colours. Mr. Murray, too, was kindness itself. There was more to point out, more people of note were present, and he showed her everything, and told her who people were, with a kindness and consideration for her ignorance which caused the girl's heart to beat high with gratitude and affection.

Of course, he knew almost everyone who was present, and he introduced her to some of his friends, and she had some very pleasant attentions paid her. Altogether she found it very delightful, and she showed plainly how she was enjoying herself.

To George the evening was a failure. He had hoped to see Miss Stanford, had almost counted upon her being here—everybody was here, and she did not come. She had stayed away on Laura's account. Mrs. Ledward had been suffering all day from a sudden and severe attack of neuralgia, and Beatrice had refused to leave her. Laura had done everything in her power to persuade her to go, but in vain. Besides, being unwilling to leave her friend and go alone, Beatrice had been far from sorry for a sufficient excuse to remain at home. She too knew that "everyone was to be there," and she guessed that George Murray would not be absent. She did not wish to see him; a meeting with him had by this time grown to be more painful than anything else. She shunned him, and had begun to be irregular in her attendance at the meetings at the Museum, while on her reading nights she came in at the last moment and left as early as she could.

George knew nothing of all this; he kept a close watch as far as he could on all new comers, hoping that each fresh couple would be Beatrice, accompanied by Mrs. Ledward, with her slender little figure and shrewd, rather sarcastic face. He kept up hope till a late hour in spite of innumerable disappointments, but by the time early people had begun to slip away he had to admit to himself that it was too late to expect her.

He was not a man to wear his heart upon his sleeve, but he could not altogether conceal that he was expectant of something, and that he was gradually making up his mind that his expectation was not going to be realised. Once he thought he saw Beatrice's figure in the distance, but when the lady came near he saw it was not she at all, and he could not altogether repress an impatient sigh. They were in the refreshment-room, and Edith looked quickly up from the ice she was eating (the first she had tasted in her life) and said—

"Mr. Murray, are you tired? Shall we go?"

"No, child, no," he said, gently; "what makes you ask that?"

"You gave such a great sigh."

"Did I," smiling. "I suppose I was thinking. Do not trouble your head about me; go on enjoying yourself."

"But I shall not enjoy myself if you don't," she said, quite gravely.

He looked down at her and smiled again, the pleasant smile which seemed to come upon his face by instinct when he was with some people, and Edith was one of them.

"You must reverse it," he said, "I brought you here to enjoy yourself, and if you don't, my mission will not be fulfilled. It is I who shall not enjoy myself if you don't; your enjoyment is mine."

She dropped her eyes, not knowing what to say, for he spoke quite seriously, and his eyes looked very kind as they rested on her face. He looked at her for what seemed to Edith quite a long time; she knew he was looking, although her own eyes were fixed upon the crimson matting which covered the floor. They were quite alone, everyone else had left the refreshment-room—even the attendants had retired into the background to have a good time amongst themselves. Edith was seated on a kind of couch against the wall, and George was near her, seated sideways on a chair, his elbow resting on the top of it and his cheek in his hand. His eyes were fixed full upon her face; yet he scarcely saw it, in spite of its delicate prettiness. His vision just now was wholly mental, and what he saw existed in his imagination alone. But Edith could not know that, and as she sat passive under his fixed gaze her heart began to beat faster than usual, and an odd feeling, which she had never experienced before, began to creep over her. She felt that her colour was growing deeper, for her face burnt hotter and hotter—she began at last to feel as if she were under a spell and would soon lose all power of motion or free will. To save herself while there was yet time, and also because she could not bear this fixed gaze any longer, she made a great effort, and suddenly stretched out her arm to put down her ice plate.

The movement broke the spell, for both herself and George. George sprang to his feet, and took the plate from her, putting it on a table near, and apologising for his negligence as he did so. Then he looked at his companion, and remarked her flushed cheeks.

"Come into one of the other rooms," he said. "It is abominably hot here; you are quite flushed; I ought not to have let you stay so long. I hope it has not made your head ache," hitting upon the feminine disorder with which he was most familiar.

"Oh no, thank you," replied Edith, "I am quite well." But she rose obediently and went out of the room with him. They walked along a corridor, and the spell being broken, the subject of their conversation come back to George's memory, and he said cheerfully,

"Now, let us go and enjoy ourselves, I can't have you looking like this; you will not care to come out with me again."

"I don't think you would miss me if I did not," she said, with an attempt at a laugh.

"You are quite mistaken," he said; "I like to have you with me very much. Now that I have tasted the joys of companionship I would not for anything be thrown back to the misery of solitary dissipation. It is much more interesting and satisfactory to have a companion, especially a sympathetic companion like you."

He looked at her again as he spoke, the same kind look which brought the colour into her cheeks, and over which no one but a little ignorant girl would have wasted any emotion.

"Do you really mean that?" asked Edith, delighted. "Do you really like to have me with you?"

"Why, yes," he answered. "I like to see you happy. I like to give you pleasure; and it is so easy to do it. To see you enjoying yourself makes me feel young again."

He spoke rather more emphatically than usual, for he felt a little remorseful. He feared lest in his absorption about Beatrice he had neglected Edith, and he took this way of making her feel that she was at present his first care.

After this they met Harry Fairfax straying about alone, and he at once accosted them, and seemed inclined to hang himself on to them. Edith was particularly glad to see him. Hitherto their meetings had been in the day-time. He had never seen her except in her simple house or street dress, never in the glory of full evening toilet, and Edith felt a girlish pride and pleasure in appearing before his dazzled vision in the soft filmy white garments which she wore this evening. He was evidently impressed by her appearance, she saw that in an instant, and was pleased by it; she was also pleased that he should find her in the company of Mr. Murray. She was therefore in very good spirits, and chatted away to him more merrily and with less shyness than he had ever known her show before, and which charmed him just as much in another way, as her quiet retiring way at home did.

George took advantage of Harry having joined them to leave Edith in his care for a few minutes while he went away on an errand which had Beatrice for its object. Harry accepted the trust with eagerness, and he and Edith spent a very pleasant ten minutes together; they talked about the pictures and the people, and Edith said a good deal about George and his kindness with which Harry chimed in; indeed he felt scarcely less grateful to George for his goodness to this lonely little girl than Edith did herself; and when George reappeared, he found the two young people evidently enjoying themselves thoroughly, so much so that he hesitated about disturbing them.

Edith felt happier than ever before in her life. The remaining half-hour of their stay, the drive home in the dark, leaning back on the soft cushions by George's side, the arrival at home, his solicitude about her having been well wrapped up, his attentions during their *tête-a-tête* supper, when he insisted upon her eating something, for she was in far too transcendental a frame of mind to think any refreshment necessary; finally her finding herself alone in her room, free to sink upon her bed and think over all the long delicious evening.

It was from this evening that the real mischief dated. The next day Edith was dreamy and only half awake, and some days passed before she was her real self again. She had begun to keep a diary when she came to Mayfield, and the entrances, at first brief, had swelled to voluminous proportions since the flower show. It took her all her spare time for two or three days to record the chronicle of this still more momentous occasion of the conversazione, with all the reflections upon and conjectures regarding the same. It took her all the longer to write because, in the midst of doing it, she kept leaning back in her chair and losing herself in dreams about it all. Over and over again she repeated to herself words which George had spoken in mere friendliness and kindness of heart, but which kept coming back to her with a significance they had never been meant to convey. She lived in them, and especially in one sentence.

George had been showing her one of the pictures, a historical subject, a scene in the life of Cromwell, and in her remarks upon it she had almost unconsciously shown a depth of ignorance which had appalled him. He had been positively silenced for a moment, and had then said gravely, but with a kindness which had taken all sting out of the words—

"You ought to know that, Edith, I do not like you to be ignorant about these things; they are what every educated Englishwoman ought to know. I should not have liked you to make that slip to a stranger. I will give you some books to read about it."

This, which to almost any other girl would have appeared a tolerably severe rebuke, seemed to poor Edith as a token of especial interest in her. She was too ignorant to be fully conscious of her ignorance, or to be ashamed of it, and she kept dwelling on these words, and saying to herself—

"He cares about it; he wants me to know more. I will read any books he likes, however dry they are. I would do anything to please him. Oh, how good he is, and how happy I am."

It is difficult to say if she was blamable or simply foolish. She was very young, very inexperienced. George Murray was the first man of his kind whom she had ever known, and he had been very kind to her, with a kindness quite different from the rough-and-ready petting she had been used to from her brothers. She had a sensitive, impressionable nature, and it was only to be expected that she should feel this kindness very deeply, and perhaps waste some emotion over it. Yet, in spite of it all, her feelings were very vague; she was certain of nothing; only her horizon had widened, and instead of life being bounded by her aunt's pleasure or displeasure, she now felt that it contained infinite possibilities. Her head was full of dreams, it is true, but they might have vanished like other dreams and left her none the worse, if it had not been for Mrs. Lester.

*(To be continued.)*

